# H2A.Z/H2B.Z double-variant nucleosomes inhabit the AT-rich promoter regions of the *Plasmodium falciparum* genome

**DOI:** 10.1111/mmi.12151

**Published:** 2013-01-28

**Authors:** Wieteke A M Hoeijmakers, Adriana M Salcedo-Amaya, Arne H Smits, Kees-Jan Françoijs, Moritz Treeck, Tim-Wolf Gilberger, Hendrik G Stunnenberg, Richárd Bártfai

**Affiliations:** 1 Department of Molecular Biology, Radboud University, Nijmegen Centre for Molecular Life SciencesNijmegen, 6525GA, the Netherlands; 2 Malaria Group, Bernhard-Nocht-Institute for Tropical MedicineHamburg, 20359, Germany; 3 Pathology and Molecular Medicine, M.G. DeGroote Institute for Infectious Disease Research, McMaster UniversityHamilton, ON, L8S 4 K1, Canada

## Abstract

Histone variants are key components of the epigenetic code and evolved to perform specific functions in transcriptional regulation, DNA repair, chromosome segregation and other fundamental processes. Although variants for histone H2A and H3 are found throughout the eukaryotic kingdom, variants of histone H2B and H4 are rarely encountered. H2B.Z is one of those rare H2B variants and is apicomplexan-specific. Here we show that in *Plasmodium falciparum* H2B.Z localizes to euchromatic intergenic regions throughout intraerythrocytic development and together with H2A.Z forms a double-variant nucleosome subtype. These nucleosomes are enriched in promoters over 3′ intergenic regions and their occupancy generally correlates with the strength of the promoter, but not with its temporal activity. Remarkably, H2B.Z occupancy levels exhibit a clear correlation with the base-composition of the underlying DNA, raising the intriguing possibility that the extreme AT content of the intergenic regions within the *Plasmodium* genome might be instructive for histone variant deposition. In summary, our data show that the H2A.Z/H2B.Z double-variant nucleosome demarcates putative regulatory regions of the *P. falciparum* epigenome and likely provides a scaffold for dynamic regulation of gene expression in this deadly human pathogen.

## Introduction

*Plasmodium falciparum* is the protozoan parasite responsible for almost 1 million malaria deaths annually (World Health Organization, 2010). It has a complex life cycle involving a mosquito vector and a human host (Bannister and Mitchell, 2003). During host infection, *P. falciparum* invades multiple different cell types and adopts various morphological states (Bannister and Mitchell, 2003), which necessitates a tight control of gene expression to allow the timely presence of key proteins (Coleman and Duraisingh, 2008). Accordingly, a cascade of gene expression has been observed during parasite intraerythrocytic development (Bozdech *et al*., 2003). However, gene regulation in *Plasmodium* is still poorly understood (Horrocks *et al*., 2009). Although, about 60 potential DNA-binding proteins are encoded in the *Plasmodium* genome (Bischoff and Vaquero, 2010), their function in gene regulation is just starting to be unveiled (Gissot *et al*., 2005; De Silva *et al*., 2008; Flueck *et al*., 2010). Besides direct regulation of gene expression by transcription factors, protein abundance can be controlled at various other levels. For example, epigenetic processes are believed to play an essential role in the regulation of transcription (Horrocks *et al*., 2009; Cui and Miao, 2010; Merrick and Duraisingh, 2010). Intriguingly, histone H3 lysine 9 acetylation (H3K9ac) marks intergenic regions (Salcedo-Amaya *et al*., 2009) and its occupancy at promoter regions positively correlates to gene expression during intraerythrocytic development (Bartfai *et al*., 2010). Furthermore, accumulating evidence indicates that epigenetic features index the *P. falciparum* epigenome into functionally distinct domains, including heterochromatic, euchromatic and centromeric regions (Flueck *et al*., 2009; Lopez-Rubio *et al*., 2009; Salcedo-Amaya *et al*., 2009; 2010; Bartfai *et al*., 2010; Hoeijmakers *et al*., 2012). Heterochromatic regions, for example, are hallmarked by the presence of H3K9 tri-methylation (H3K9me3) (Lopez-Rubio *et al*., 2009; Salcedo-Amaya *et al*., 2009) and heterochromatic protein 1 (HP1) (Flueck *et al*., 2009), whereas euchromatic regions were previously shown to possess H3K4me3 and H3K9ac marks as well as histone variant H2A.Z (Salcedo-Amaya *et al*., 2009; Bartfai *et al*., 2010).

Epigenetic mechanisms act at the level of chromatin and influence all DNA-associated processes in a cell (Felsenfeld and Groudine, 2003). The nucleosome that forms the building block of chromatin is composed of an octameric protein core containing two copies each of the core histones (H2A, H2B, H3 and H4) encircled by ∼ 147 bp of DNA (Luger *et al*., 1997). The N-terminal histone tails that protrude from this core can be post-translationally modified at various residues, thereby influencing nucleosomal properties and function (Felsenfeld and Groudine, 2003). In addition, core histones can be exchanged by histone variants, which have a somewhat different amino acid sequence and can bear distinct post-translational modifications. Histone variants play various functions in different organisms and are often essential (Brown, 2001; Pusarla and Bhargava, 2005).

The *P. falciparum* genome encodes four histone variants (H2A.Z, H3.3, CenH3 and H2Bv, which has recently been renamed to H2B.Z) (Miao *et al*., 2006; Talbert *et al*., 2012) for two of which we recently revealed their genome-wide localization. Similar to its conserved function in other organisms (Dalal, 2009), *Pf*CENH3 was found to demarcate *P. falciparum* centromeres (Hoeijmakers *et al*., 2012). In contrast, histone variant H2A.Z has been reported to be involved both in gene activation and in gene silencing in different organisms and localizes to various distinct genomic features, including gene promoters and heterochromatin boundaries (Talbert and Henikoff, 2010). In *P. falciparum*, this histone variant occupies euchromatic intergenic regions of the genome, which are involved in transcriptional control (Bartfai *et al*., 2010; Petter *et al*., 2011). In contrast, the function of histone variant *Pf*H2B.Z has not been explored.

Contrary to the various distinct variants of histone H2A and H3 that are found throughout the eukaryotic kingdom, variants of histone H2B and H4 are rarely observed (Talbert and Henikoff, 2010). H2B.Z is an apicomplexan-specific histone variant of histone H2B and has evolved independently of the H2B variant of trypanosomid parasites, H2BV (Dalmasso *et al*., 2011). Notwithstanding their different evolutionary origin, in the apicomplexan parasite, *Toxoplasma gondii* (Dalmasso *et al*., 2006; 2009) and in the trypanosomatid *Trypanosoma brucei* (Lowell *et al*., 2005) these H2B variants dimerize with histone variant H2A.Z and associate with active histone marks (Mandava *et al*., 2008; Dalmasso *et al*., 2009; Siegel *et al*., 2009). Furthermore, genome-wide analyses in *T. brucei* revealed that H2BV is enriched on transcription start sites of polycistronic transcripts (Siegel *et al*., 2009).

As a first step towards identification of H2B.Z function in *Plasmodium*, we set out to unravel the genome-wide localization of *P. falciparum* H2B.Z. We show that histone variants H2A.Z and H2B.Z colocalize to euchromatic intergenic regions and reside in a double-variant nucleosome subtype. Collectively, our data indicate that these double-variant nucleosomes demarcate regulatory regions of the *P. falciparum* genome and might play a role in transcriptional control of this important human pathogen.

## Results

### *Pf*H2B.Z localizes to euchromatic intergenic regions of the *P. falciparum* genome

**Fig. 1:**
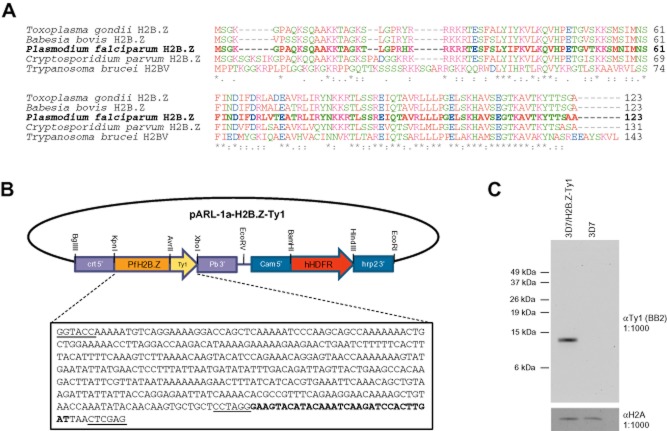
Generation of H2B.Z-Ty1 3D7 parasite line. A. Alignment of the amino acid sequence of H2B variants in various protozoan parasites. *Plasmodium falciparum* H2B.Z (PF3D7_0714000), *Toxoplasma gondii* H2B.Z (TGME49_009910), *Babesia bovis* H2B.Z (BBOV_IV006840), *Cryptosporidium parvum* H2B.Z (cgd7_1700) and *Trypanosoma brucei* H2BV (Tb11.02.5250) are included in this analysis. Amino acid identity is indicated by asterisk (*) and similarity by dots (. or :) and colour code. B. Map of the pARL-1a-H2B.Z-Ty1 plasmid transfected into 3D7 *P. falciparum* parasites. Restriction sites are underlined and Ty1-tag sequence is indicated in bold. C. Western blot of acid-extracted histones demonstrating specific recognition of H2B.Z-Ty1 by the BB2 antibody in the transgenic parasite line.

*Plasmodium falciparum* histone variant H2B.Z (PF3D7_0714000) encodes for a 123-amino-acid protein that differs from canonical H2B (PF3D7_1105100) mostly in its N-terminal domain (Miao *et al*., 2006). Sequence comparison (Fig. 1A) reveals that *Pf*H2B.Z is similar to other apicomplexan histone H2B variants (89%, 86% and 79% identity or 99%, 96% and 98% similarity to *T. gondii*, *Babesia bovis* and *Crytosporidium parvum* respectively), but differs substantially from *T. brucei* H2B variant H2BV (only 37% identity and 81% similarity).

In order to characterize this histone variant in *P. falciparum*, we generated a parasite line (3D7) carrying an episomal Ty1-tagged copy of *Pf*H2B.Z (Fig. 1B). Western blot analysis of protein extracts from the transgenic parasite line and from the 3D7 mother line revealed the expression of the tagged protein at the expected size (14 kDa) and its specific recognition by the anti-Ty1 antibody (Fig. 1C).

**Fig. 2:**
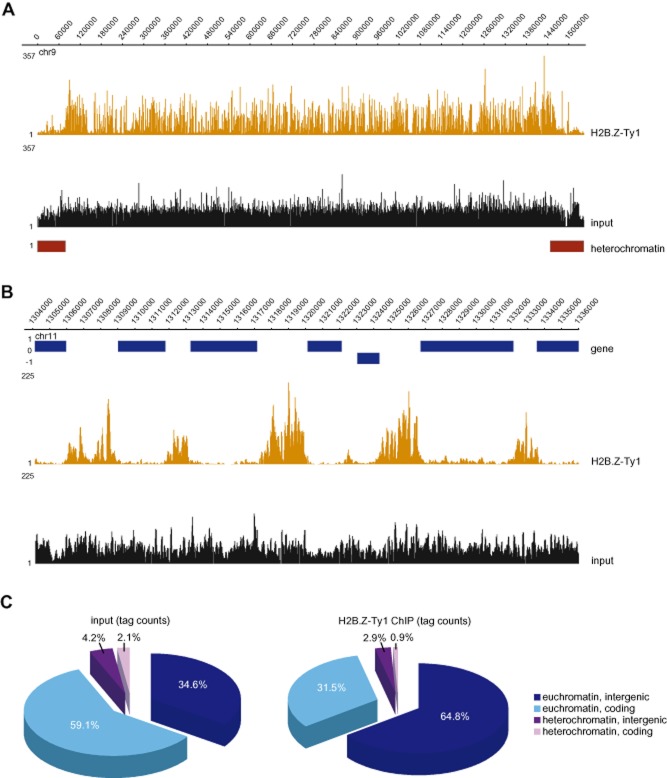
*P. falciparum* H2B.Z localizes to euchromatic intergenic regions. A and B. Coverage plot of H2B.Z-Ty1 and corresponding input distribution over the entire chromosome 9 (A) and part of an euchromatic region at chromosome 11 (B) obtained from schizont-stage parasites. Heterochromatin is indicated in dark red and genes are depicted as blue boxes. C. Piecharts plotting the proportion of sequence reads mapped on euchromatic intergenic or coding and heterochromatic intergenic or coding regions of H2B.Z-Ty1 ChIP-Seq and input from schizont-stage parasites.

Native chromatin immunoprecipitation (nChIP) of mono-nucleosomal DNA fragments, followed by linear amplification for deep sequencing (LADS) (Hoeijmakers *et al*., 2011) and Illumina sequencing was performed to obtain a minimum of 10.6 M 76 bp sequence reads for both H2B.Z-Ty1 ChIP and corresponding input. Visual inspection of H2B.Z-Ty1 coverage plots of schizont-stage parasites reveals a clear enrichment of signal in euchromatic regions of the genome (Fig. 2A), which are depleted for the heterochromatic H3K9me3 mark (Lopez-Rubio *et al*., 2009; Salcedo-Amaya *et al*., 2009) as well as heterochromatic protein 1 (Flueck *et al*., 2009). Zooming into the euchromatic domain, a specific localization to intergenic regions can be observed (Fig. 2B). Counting the number of sequence reads localizing to euchromatic coding, euchromatic intergenic, heterochromatic coding or heterochromatic intergenic regions in ChIP and input libraries, respectively, corroborated the preferential localization of histone variant H2B.Z to euchromatic intergenic regions (Fig. 2C).

### *Pf*H2B.Z and *Pf*H2A.Z colocalize and form a double-variant nucleosome subtype

**Fig. 3:**
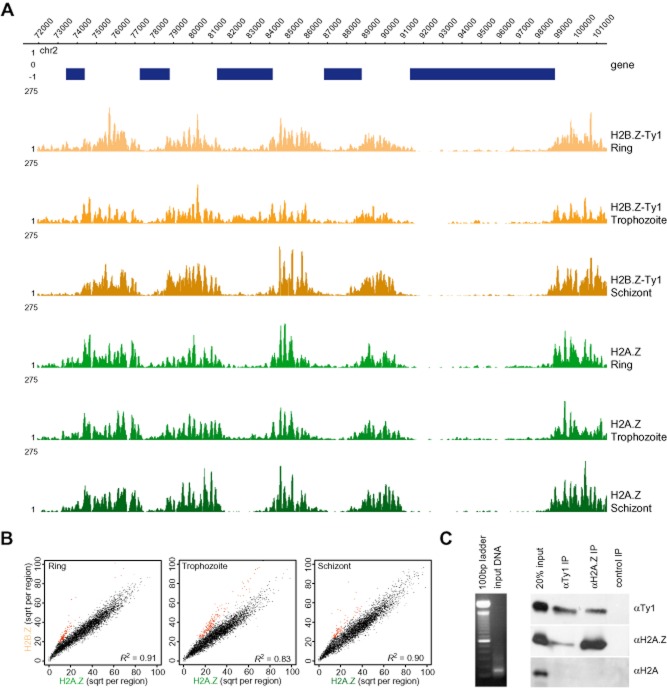
*Pf*H2A.Z and *Pf*H2B.Z have similar genome-wide localization profiles throughout intraerythrocytic development and reside in the same nucleosome particle. A. Screenshot of the H2B.Z-Ty1 and H2A.Z ChIP-Seq coverage plots over a part of an euchromatic region of *P. falciparum* chromosome 2 at ring, trophozoite and schizont-stage parasites. Genes are depicted as blue boxes. B. Scatterplot analysis of H2A.Z and H2B.Z-Ty1 ChIP-Seq sequence reads mapped on intergenic regions of the entire genome, obtained from ring, trophozoite and schizont parasites. *R*-square values are included in each graph. C. Co-immunoprecipitation experiment performed on mono-nucleosomes isolated from H2B.Z-Ty1 parasites. Left panel: DNA isolated from input material used for immunoprecipitation as resolved on 1.5% agarose gel. Right panel: Western blot analysis of histone proteins (H2A, H2A.Z and H2B.Z-Ty1) (co)immunoprecipitated by the anti-H2A.Z, anti-Ty1 and a control antibody.

The genome-wide profile of H2B.Z-Ty1 at schizont stage reveals a localization very similar to what we previously observed for histone variant *Pf*H2A.Z (Bartfai *et al*., 2010). To examine whether the co-occupancy of these two histone variants is stable throughout intraerythrocytic development, H2B.Z-Ty1 occupancy was also determined in ring and trophozoite stages. Indeed, both histone variants clearly occupy euchromatic intergenic regions at all three stages of development (Fig. 3A, Supplementary Fig. S1). Furthermore, similar to H2A.Z, the level of H2B.Z occupancy remains largely unchanged during intraerythrocytic development (Fig. 3A, Supplementary Fig. S1) and does not correlate to the temporal activity of nearby genes (Supplementary Fig. S2).

When comparing H2A.Z and H2B.Z-Ty1 tag counts in intergenic regions (Fig. 3B), a very strong positive correlation can be observed, suggesting the colocalization of both histone variants over the *Plasmodium* genome. A closer examination of the scatterplots (Fig. 3B) reveals a few regions indicated in red that display higher signals in the H2B.Z-Ty1 experiment compared with the H2A.Z profiles. Most of these signals were found to originate from heterochromatic domains (data not shown), which might indicate that heterochromatic intergenic regions contain low levels of histone variant H2B.Z especially at trophozoite stages. Notably, the trophozoite H2B.Z-Ty1 dataset also displays slightly lower enrichment at intergenic regions and provide signal in coding bodies of genes. This can be observed in the coverage plot (Fig. 3A) and the reduced correlation between H2B.Z and H2A.Z intergenic signals in trophozoite stage parasites (Fig. 3C). Although this slightly different pattern at the trophozoite stage is also apparent in a biological replicate (targeted ChIP, data not shown), it remains ambiguous whether it is a true biological phenomena or due to the ectopic (miss)expression of the tagged H2B.Z protein.

The colocalization of both variants in *P. falciparum* suggests that H2A.Z and H2B.Z variants reside in the same nucleosome particle. For that reason, we performed an immunoprecipitation experiment on mono-nucleosomes derived from the H2B.Z-Ty1 line (Fig. 3C, left panel). Immunoprecipitation using αH2A.Z antibody results in pull-down of H2B.Z-Ty1, and similarly immunoprecipitation of H2B.Z-Ty1 results in co-IP of H2A.Z. (The seeming ratio-difference between H2A.Z and H2B.Z co-precipitation in the IPs can be largely explained by the fact that only a fraction of the total H2B.Z pool carries the Ty1-tag – resulting in visualization of only part of the H2B.Z co-immunoprecipitated with H2A.Z on Western blot – as well as the relatively poor IP efficiency of the αTy1 antibody.) Notably, neither αH2A.Z nor the αTy1 antibody immunoprecipitated substantial amounts of H2A. However, given the low affinity of the H2A antibody we cannot exclude the possibility that in some of these nucleosomes canonical H2A protein is present (e.g. H2B.Z/H2A). Importantly, a control IP using control rabbit IgG co-immunoprecipitates neither of the histones (Fig. 3C, right panel).

In summary, our analysis reveals that *P. falciparum* H2B.Z and H2A.Z histone variants primarily reside in the same nucleosome particle and demarcate euchromatic intergenic regions throughout the intraerythrocytic development.

### *Pf*H2B.Z and *Pf*H2A.Z preferentially localize to AT-rich promoter regions

**Fig. 4:**
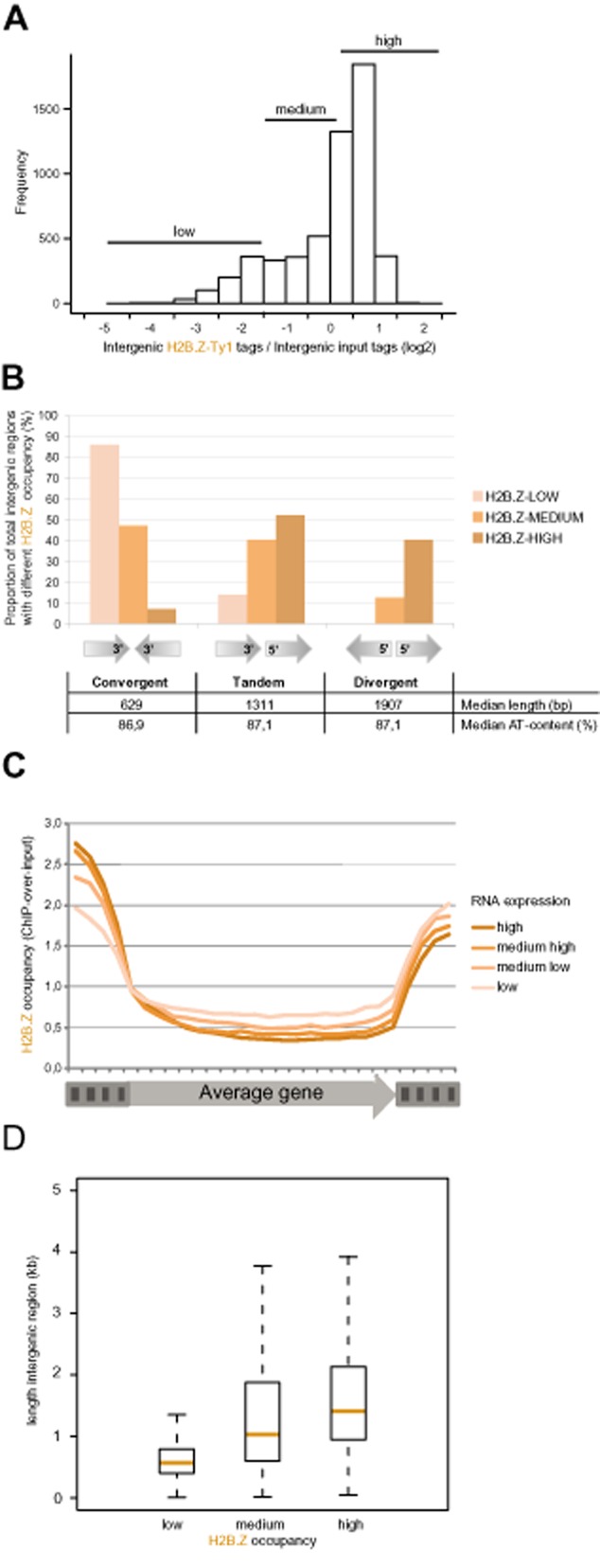
*Pf*H2B.Z occupancy is enriched in long promoter-containing intergenic regions. A. Distribution of H2B.Z-Ty1 ChIP-over-input log2-ratios of intergenic regions of the *Plasmodium* genome, displayed as histogram and divided into three categories (low, medium, high) based on H2B.Z occupancy. Note that both euchromatic and heterochromatic intergenic regions are included in this analysis. B. Histogram displaying the percentage of euchromatic intergenic regions with low, medium or high H2B.Z occupancy in schizont-stage parasites with respect to the orientation of their neighbouring genes [convergent (3′–3′), tandem (3′–5′) or divergent (5′–5′)]. Table displays the median length and AT content of euchromatic intergenic regions in the above categories. C. Average profile of ChIP-over-input ratios of H2B.Z-Ty1 occupancy showing the distribution of this variants over total 1855 euchromatic genes, divided to four groups with low, medium low, medium high or high expression levels [based on earlier RNA-seq data (Bartfai *et al*., 2010)]. Coding body is indicated by an arrow (divided into 20 bins), 5′ and 3′ intergenic regions are displayed as four blocks of 150 bp. The profiles have been generated from the ring-stage dataset where the correlation between H2B.Z occupancy at promoters and gene expression is most prevalent. D. Box plots displaying the distribution of H2B.Z-Ty1 ChIP-over-input ratios in low, medium and highly H2B.Z-occupied regions plotted against the length of the euchromatic intergenic region.

Although H2B.Z is enriched in almost every euchromatic intergenic region, its occupancy differs considerably between individual regions (divided to low, medium or high on Fig. 4A). Most intergenic regions with low H2B.Z occupancy reside in the heterochromatic domain, but such regions also occur within the euchromatin. Additionally, intergenic regions displaying medium as well as high H2B.Z occupancy inhabit the euchromatic domain (Supplementary Fig. S3). To explore the variable levels of H2B.Z in different euchromatic intergenic regions, we investigated which genomic features correlate with variant histone occupancy.

Visual inspection of the occupancy profiles suggest that regions upstream of genes maintain higher variant histone levels as compared with the intergenic regions downstream of genes. Indeed, when categorizing euchromatic intergenic regions based on the orientation of adjacent genes either as convergent (neighboured by two 3′ ends), tandem (neighboured by one 3′ and one 5′ end) or as divergent (neighboured by two 5′ ends) high occupancy is most prevalent in promoter containing regions (tandem or divergent). On the contrary, low H2B.Z occupancy is almost exclusively observed at convergent regions (two 3′ ends) (Fig. 4B). This finding indicates that although H2B.Z-containing nucleosomes are generally enriched in euchromatic intergenic regions, occupancy is highest at promoter regions of genes. Higher H2B.Z occupancy at promoter regions could also be observed on the average gene profile (Fig. 4C). Furthermore, from the average H2B.Z profiles computed for genes with different expression levels it also became obvious that H2B.Z enrichment at the promoter is most pronounced on the most abundantly expressed genes (Fig. 4C) indicating that H2B.Z occupancy correlates with the strength of the promoters. Interestingly, the promoter-containing intergenic regions of the *Plasmodium* genome appear to be on average three times longer than 3′ intergenic regions (Fig. 4B – table), which results in a strong positive correlation between H2B.Z occupancy and the length of euchromatic intergenic regions (Fig. 4D).

**Fig. 5:**
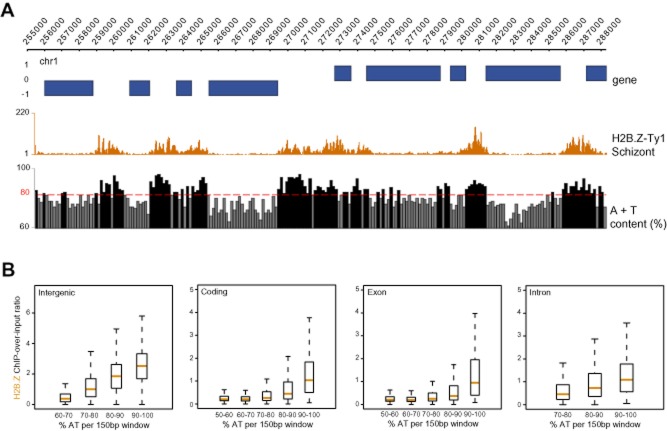
*Pf*H2B.Z occupancy increases with genomic AT content. A. Screenshot of H2B.Z-Ty1 coverage plot illustrating an increase in variant occupancy on high AT-rich regions. 150 bp windows with more than 80% AT (red dotted line) are indicated as black bars. B. Box plots displaying the distribution of H2B.Z-Ty1 ChIP-over-input ratios in 150 bp windows of euchromatic intergenic, coding, exonic and intronic regions plotted against their genomic AT content. All analyses were performed on data obtained from schizont-stage parasites.

Visual inspection of the coverage plots furthermore suggested that H2B.Z is primarily present at regions with AT content higher than 80% (Fig. 5A). To explore the possibility that genomic AT content was correlative to histone variant occupancy, we calculated the ChIP-over-input ratio for every 150 bp window of the euchromatic domain for H2B.Z-Ty1 as well as H2A.Z and plotted it against their AT content. As shown in the box plots in Fig. 5B and Supplementary Fig. S4A, intergenic regions with increasing AT content indeed display markedly increased median H2B.Z and H2A.Z occupancies. Interestingly, although H2B.Z and H2A.Z are primarily present in intergenic regions of the genome, genic regions (both exons and introns) with extreme AT content also exhibit elevated variant histone levels (Fig. 5B, Supplementary Fig. S4B) although not to the extent of intergenic regions. To explore the possibility that preferential localization of H2B.Z or H2A.Z towards promoter regions might be guided by an increased AT content in these domains, we calculated the average AT content for convergent, divergent and tandem intergenic regions (Fig. 4C – table). However, the median AT content of these different regions is virtually identical, showing either that the preferential localization of H2B.Z/H2A.Z to promoter containing and AT-rich intergenic regions is driven by independent mechanisms or that efficiency of double-variant nucleosome deposition depends on the size of the AT-rich region (considering the reduced length of 3′ compared with 5′ intergenic regions – Fig. 4B).

In conclusion, we have shown that histone variants H2A.Z and H2B.Z form a double-variant nucleosome subtype and locate to euchromatic intergenic regions of the *P. falciparum* genome where they display highest occupancies in promoter regions. Furthermore, both H2B.Z and H2A.Z histone variant occupancy displays a clear positive correlation towards genomic AT content.

## Discussion

In this study we show that histone variants H2A.Z and H2B.Z can reside in a double-variant nucleosome subtype in the human malaria parasite *P. falciparum*, similar to *T. gondii* H2A.Z/H2B.Z (Dalmasso *et al*., 2009) and *T. brucei* H2A.Z/H2BV nucleosomes (Lowell *et al*., 2005). We reveal that both variants localize to euchromatic intergenic regions throughout intraerythrocytic development and are enriched on promoters over 3′ intergenic domains. Furthermore, H2A.Z and H2B.Z levels increase on longer intergenic regions and show a remarkable positive correlation to genomic AT content.

*Pf*H2B.Z and *Pf*H2A.Z ChIP-seq localization profiles both display clear occupancy of euchromatic intergenic regions throughout intraerythrocytic development, demonstrating that H2B.Z/H2A.Z double-variant nucleosomes occupy intergenic regions of the *Plasmodium* genome in a rather ubiquitous fashion. We did observe some minor differences between H2A.Z and H2B.Z levels especially in the heterochromatic domain and in particular at the trophozoite stage. However, we cannot exclude that this discrepancy is at least partially resulting from slight over- and/or misexpression of the H2B.Z-Ty1 protein. Similarly, the generally lower enrichment of H2B.Z-Ty1 in intergenic regions compared with H2A.Z enrichment could be a technical issue as not every H2B.Z histone is expected to carry the Ty1 tag in these parasites and therefore only part of the H2B.Z pool will be precipitated. Notwithstanding these minor quantitative differences, H2A.Z and H2B.Z clearly colocalizes at most part of the genome and are continuously present in the vast majority of intergenic regions throughout intraerythrocytic development. Given that H3K4me3 and H3K9ac marking occurs in the same regions, but their levels vary during intraerythrocytic development (Bartfai *et al*., 2010), it seems plausible that H2A.Z and/or H2B.Z is specifically recognized by writer, reader and/or eraser complexes placing/removing these marks. Further studies to unravel which components of this ‘scaffolding’ nucleosome might be specifically recognized by different epigenetic complexes will be necessary to identify the molecular mechanisms underlying the dynamic histone modifications found associated with euchromatic intergenic domains.

Interestingly, deletion of the first 23 amino acids of the H2BV N-terminal tail in *T. brucei* did not affect parasite viability, contrary to deletion of full-length H2BV which is essential (Lowell *et al*., 2005). This suggests that this part of the tail of the H2BV protein as well as the acetylations found on K7 and K19 (Mandava *et al*., 2008) are dispensable for recognition of this nucleosome subtype for essential functions. However, it remains to be tested whether H2B.Z, H2A.Z or their N-terminal tails are dispensable for survival of *P. falciparum*. Interestingly, the N-terminal tail of *P. falciparum* H2A.Z as well as *Pf*H2B.Z can be highly acetylated at several positions (Miao *et al*., 2006; Trelle *et al*., 2009). The functional relevance of the observed modifications is, however, completely unexplored. Furthermore, it is also unclear whether H2B.Z and/or H2A.Z acetylation in *Plasmodium* is dynamic, whereby it might contribute to dynamic recruitment of epigenetic enzymes to the nucleosome ‘scaffold’. Alternatively, H2A.Z and/or H2B.Z acetylation could influence nucleosome properties. Experimental data in *T. brucei* (Siegel *et al*., 2009) and in *P. falciparum* (R. Bártfai, W. Hoeijmakers and H. Stunnenberg, unpubl. obs.) indicate that variant nucleosomes in both species are less stable compared with their canonical counterparts. Consequently, double-variant nucleosomes residing on regulatory DNA sequences might be more readily displaced by chromatin remodelers or transcription machinery. Alternatively, these nucleosomes might display a reduced residence time (e.g. a higher ‘off’ rate) on the DNA, thereby temporally exposing regulatory sequences for transcription factor binding. In either of these models, the H2A.Z/H2B.Z double-variant nucleosome subtype likely plays a supportive function in transcriptional initiation in line with their increased levels on promoter regions (Fig. 4C) and the higher H2A.Z/H2B.Z occupancy identified on strong promoters (fig. S11 of Bartfai *et al*., 2010 and Fig. 4D).

In this study we observe a clear enrichment of H2B.Z in long promoter containing intergenic regions over 3′ UTR containing regions which are considerably shorter (Fig. 4). A simple mechanism underlying this observation could be that incorporation of H2B.Z to promoter regions is coupled to the transcription initiation machinery. However, in this scenario one would expect that H2B.Z occupancy would vary with temporal activation of the gene, which is clearly not the case. Alternatively, the elongating transcriptional machinery might evict H2B.Z-containing nucleosomes, which are then replaced by canonical nucleosomes. The effect of this would be a lower H2B.Z occupancy in the coding body and UTR regions and could be more pronounced in shorter 3′ UTR containing intergenic regions. However, in this scenario, ring stage-specific genes should display H2B.Z marking in the schizont stage (after replication, but before gene expression), which is not the case. Alternatively, the markedly lower H2B.Z occupancy in short 3′ UTR containing intergenic regions could potentially indicate that a minimal length of the (AT-rich) intergenic region *per se* might be required to trigger efficient deposition of H2A.Z/H2B.Z-type nucleosomes.

Besides the abovementioned correlation, we have observed an even more striking correlation between H2B.Z and H2A.Z occupancy and genomic AT content. Importantly, this correlation is not simply the reflection of the different AT content of coding and intergenic regions, but could be observed within the intergenic region as well as in coding bodies of genes. One possible explanation for this observation would be that H2A.Z/H2B.Z nucleosomes ‘prefer’ highly AT-rich regions. Occupancy by canonical nucleosomes is reported to be elevated in GC-rich regions in various *in vitro* and *in vivo* studies (Kaplan *et al*., 2010) and the presence of poly-dAdT or long homopolymeric A or T stretches have been reported to be unfavourable for nucleosome formation (Kaplan *et al*., 2009; Segal and Widom, 2009). Given the strong correlation between double-variant nucleosome occupancy and AT richness, it is tempting to speculate that H2A.Z/H2B.Z nucleosomes might posses an intrinsic preference for highly AT-biased genomic DNA. Indeed, an altered DNA sequence specificity for certain histone variants has been suggested recently (Segal and Widom, 2009). Interestingly, the sequence preference of H2A.Z-containing nucleosomes was shown to be very similar to that of canonical nucleosomes in budding yeast (Albert *et al*., 2007). Accordingly such preference for high AT content might require the presence of H2B.Z. Alternatively, the higher occupancy of double-variant nucleosomes at AT-rich regions might be driven by a histone chaperone with a preference for AT-rich sequences depositing H2A.Z/H2B.Z dimers preferentially at these sites.

Moreover, we observed a moderate, but clear positive trend between the AT content of euchromatic promoter regions and the maximum level of gene expression during intraerythrocytic development (Supplementary Fig. S5). This might indicate that genomic AT content influences promoter strength. This is particularly interesting, considering that H2B.Z occupancy upstream of genes positively correlates to the expression level of these genes (Fig. 4C) and might suggest that higher AT content and variant histone occupancy leads to increased promoter strength. Targeted experiments testing the above hypotheses will be pertinent to reveal the mechanisms underlying these extraordinary correlations.

As described above, double-variant nucleosomes are generally absent from the heterochromatic domain and within the euchromatic regions their level is largely invariable between the stages of intraerythrocytic development. Intriguingly, H2A.Z/H2B.Z nucleosomes also ubiquitously occupy the highly AT-rich introns of antigenic variation (*var*) genes (Bartfai *et al*., 2010; Petter *et al*., 2013 article in the same issue of *Molecular Microbiology*, data not shown) further supporting the hypothesis on the AT content driven deposition of these nucleosomes. On the contrary, however, dynamic deposition of H2A.Z (Petter *et al*., 2011) and H2B.Z (Petter *et al*., 2013 article in the same issue of *Molecular Microbiology*) has been observed at the promoter region of the single active *var* gene. This stage-specific deposition of double-variant nucleosomes associated with the temporal activity of this active *var*. Therefore, it seems that although AT content might act as a targeting mechanism for H2A.Z/H2B.Z nucleosomes, other mechanisms are in place to fine-tune and modulate the localization pattern of this special nucleosome subtype.

In light of the increasing drug resistance against current anti-malarial medication, there is an urgent need to identify new targets for therapeutic intervention. Processes that are specific to the parasite, when compared with its human host provide targets for drug development. As H2B.Z is an apicomplexan-specific epigenome component, processes targeting this variant could provide a promising target for development of anti-malarial drugs. Our study suggests that *P. falciparum* H2B.Z may play a role in epigenome organization and gene expression regulation by demarcation of regulatory elements in the *Plasmodium* genome. Consequently, enzymes targeting H2B.Z-containing nucleosomes might be candidates for drug development. Insight in the preferential deposition or recognition of H2B.Z nucleosomes by effector–protein complexes is currently lacking. Therefore, additional studies to unravel the properties of H2A.Z/H2B.Z double-variant nucleosomes, identify the chaperone involved in H2B.Z deposition and uncover effector–proteins that specifically recognize this nucleosome subtype will be imperative to explore the potential use of epidrugs targeting H2B.Z-associated pathways in the battle against malaria.

## Experimental procedures

### Parasite culture

Parasites were cultured in standard RPMI medium supplemented with 10% human serum and 0.2% NaHCO3 and 2.5% human O^+^ red blood cells in 250 ml tissue culture flasks and candle jars. The culture was synchronized with multiple rounds of sorbitol treatments and Percoll gradient centrifugation. A last Percoll gradient centrifugation was applied in the cycle prior to collection as in Bartfai *et al*. (2010). Medium was changed at every 10 h, but not less than 10 h before collection. After 20 h post invasion (hpi), double volume of medium was added to ensure optimal development of the parasites. The culture was divided to separate culture flasks (20 ml each) but these were mixed with every change of medium. Ring, trophozoite or schizont-stage parasites were collected 20, 30 or 40 hpi respectively.

For profiling of H2B.Z, 3D7 *P. falciparum* parasites were transfected with a pARL-1a- plasmid (Crabb *et al*., 2004) encoding a C-terminally Ty1-tagged version of *Pf*H2B.Z (PF3D7_071400) under the control of the chloroquine resistance transporter promoter (Fig. 1B). Expression of the transgene in the presence of 20 nM WR99210 was confirmed using the monoclonal anti-Ty1 antibody (BB2) that exclusively recognizes the tagged H2B.Z protein on Western blot (Fig. 1C).

### Chromatin immunoprecipitation

Native ChIP was carried out essentially as described in Bartfai *et al*. (2010). In short: red blood cells and parasites were lysed using saponin and a hypotonic buffer, after which nuclei were separated using a 0.25 M sucrose buffer cushion. Native chromatin was prepared by MNase digestion and subsequent extraction with salt-free buffers (10 mM Tris pH 7.4, 1 mM EDTA; and 1 mM Tris pH 7.4, 0.2 mM EDTA). Chromatin was diluted in 2× ChIP incubation buffer (100 mM NaCl, 20 mM Tris pH 7.4, 6 mM EDTA, 1% Triton X-100, 0.1% SDS). Approximately 400 ng of DNA-containing chromatin was incubated with 2 μg of BB2 antibody overnight at 4°C followed by the addition of 10 μl A/G beads (SantaCruz Biotechnology) and further incubated for 2 h. After washing with buffers containing 100, 150 and 250 mM NaCl, immunoprecipitated DNA was eluted and purified using PCR purification columns (Qiagen). Five ChIP reactions were performed in parallel and pooled to obtain sufficient amount of DNA for ChIP-seq.

### High-throughput sequencing

Sequencing libraries were prepared from 6.1–40 ng of H2B.Z-Ty1 ChIP or corresponding mono-nucleosomal input DNA as described in Hoeijmakers *et al*. (2011). Sequencing libraries (14–15 pmol each) were loaded on the Illumina Genome Analyser IIx and sequenced for 76 cycles from one side of the fragments (Standard Cluster Generation Kit v4 and 2 × 36-cycle sequencing kit v4). Seventy-six-base-pair sequence reads were mapped against the *P. falciparum* genome assembly (PlasmoDB v6.1) using BWA (Li and Durbin, 2009).

### Data analysis

*Pf*H2A.Z genome-wide localization profiles were used from Bartfai *et al*. (2010). For comparative analysis 10.6 M uniquely mapped reads were randomly selected for each dataset. As the H2B.Z-Ty1 parasite line carried multiple deletions in its subtelomeres at chromosomes 1, 4 and 7 (positions 558360–643292, 6800–33800 and 1437250–1501717 respectively) and a single duplication at chr. 13 (position 150901–380691), these regions were excluded in all subsequent analysis.

Coverage plots were generated by counting the number of overlapping, uniquely mapped 76 bp tags in 10 bp windows and visualized in SignalMap (NimbleGen). Coverage plots shown in Figs 2, 3 and 5 and Supplementary Fig. S1 are based on 5.5 M uniquely mapped reads for both histone variants and ∼ 10 M uniquely mapped reads for the input samples. Generation of mappability tracks is explained in more detail in Bartfai *et al*. (2010). For all subsequent analysis tags were assigned to a particular region if the middle position of the 147 bp mono-nucleosomal fragment falls into this region. Heterochromatin was defined by the presence of H3K9me3 (Salcedo-Amaya *et al*., 2009) and HP1 (Flueck *et al*., 2009). Regions localized on the hetero-euchromatic border were excluded from the analysis.

For scatterplot analysis, the number of uniquely mapped tags were counted in each intergenic region of the H2B.Z-Ty1 genome. H2B.Z square-rooted signals were plotted against H2A.Z square-rooted occupancy values in R for ring, trophozoite and schizont stages. *R*^2^ of square-rooted values were determined in R using the ‘lm’ function.

Regions of low-, medium- or high-histone variant occupancy were defined as in Bartfai *et al*. (2010) by calculation of the ChIP-over-input ratio for each intergenic region of the H2B.Z-Ty1 genome and subsequent plotting as histogram in R (Fig. 4A). H2B.Z low = −4.5 to −1 (log2), H2B.Z medium = −1 to 0.75 (log2) and H2B.Z high = 0.75 to 3 (log2). Euchromatic intergenic regions were classified as convergent (containing two 3′ UTRs), tandem (containing one 3′ and one 5′ UTR) or divergent (containing two 5′ UTRs) and the number of H2B.Z low, medium- or high-occupancy regions were counted for each class. Subsequently, the percentage of total low-, medium- or high-occupancy regions allocated to each intergenic class were plotted in Fig. 4C. The sequence composition and length of these regions were calculated by self-written scripts and Excel.

Average gene profiles were computed for 1855 genes within the euchromatic domain (given the general lack of signal in the heterochromatic domain) with coding body of 1–10 kb (excluding extremely small or large genes) and a minimum of 800 bp intergenic regions on both sides (to minimize overlap with flanking region of nearby genes) as in Bartfai *et al*. (2010). Genes were categorized based on their expression level at late ring stage [number of RNAseq tags per kb of coding regions in Bartfai *et al*. (2010)] to four quartiles (low: 1–26; medium low: 27–182; medium high: 183–671; and high: 672–91262). ChIP-over-input ratios were determined for each of the 20 bins within the coding body of genes and each of the four 150 bp windows immediately flanking the coding sequence. The average ChIP-over-input ratio for genes with different expression levels was plotted in Fig. 4C.

Box plots were generated in R using standard settings (outlier values were not shown). For Fig. 4D, the length of each euchromatic intergenic region assigned as low, medium or high H2B.Z (see Fig. 4A for category definition) was plotted.

To determine a correlation between H2B.Z or H2A.Z occupancy and genomic AT content (Fig. 5B), ChIP-over-input ratios were determined for every 150 bp window in euchromatic intergenic, euchromatic coding, euchromatic exonic or euchromatic intronic regions (regions below 100 bp in length or with a zero input value for any of the stages were excluded from the analysis). AT content for each 150 bp window was determined and H2B.Z/H2A.Z ChIP-over-input ratios of ring, trophozoite and schizont-stage parasites were plotted as box plot for each AT category (50–60% AT, 60–70% AT, 70–80% AT, 80–90% AT and 90–100% AT) in R (unless otherwise indicated, only schizont-stage data are shown in the figures). AT categories containing less than forty 150 bp windows were not included in the plots.

For Supplementary Fig. S2 the heatmaps were generated by MultiExperimentViewer as described earlier (Bartfai *et al*., 2010). In short, temporal transcriptional activity of each euchromatic gene was calculated across eight stages of intraerythrocytic development and were used to divide genes to 12 groups based on their expression pattern (k-means clustering). The expression profile of each gene was then matched with the profile of H3K9ac marking or H2A.Z/H2B.Z occupancy in the H2A.Z occupied region (peak) immediately upstream of the gene. Relative marking/occupancy through the intraerythrocytic cycle was calculated similarly to that of the relative transcriptional activity (scaled to the sum of ChIP-over-input ratios across stages). For H2B.Z relative occupancy values were divided by 1.33 given that the analysis was performed in three instead of four stages.

To define the correlation between AT content of the promoter region and gene expression, the AT content of the 600 bp sequence upstream of 1855 euchromatic genes (the same as used for the generation of average gene profiles) was defined by a self-written script. This was correlated to the maximum expression level of the gene across 8 stages intraerythrocytic development [defined by the highest number of RNAseq reads per 1000 bp coding sequence (Bartfai *et al*., 2010)]. Scatterplot and table were generated using Excel.

### Western blotting

Acid-extracted histones from 3D7 mother and H2B.Z-Ty1 transgenic line were separated on 18% SDS-PAGE and transferred to Protean nitrocellulose membrane (Whatman, 0.45 μm). The membrane was probed with mouse αTy1 (1:1000, BB2 antibody) and rabbit αH2A (1:1000, Millipore 07-146) and secondary αMouse IRDye 680 (1:15000, LI-COR Biosciences 926-32220) and αRabbit IRDye 800CW (1:15000, LI-COR 926-32211) and measured on the Odyssey system (LI-COR Biosciences). Co-immunoprecipitations were performed on mono-nucleosomes harvested from H2B.Z-Ty1 schizont stages as for native ChIP using 2 μg of BB2, 1 μl of α*Pf*H2A.Z (from Bartfai *et al*., 2010) or 1 μg of control rabbit IgG [Upstate (Millipore) 12-370]. Three ChIP reactions were performed for each antibody, proteins were precipitated by acetone and pooled for Western analysis (as above) together with half an input reaction. The membrane was probed with mouse αTy1 (1:1000, BB2 antibody), rabbit α*Pf*H2A.Z (1:2500; Bartfai *et al*., 2010) or rabbit αH2A (1:1000, Millipore 07-146) and secondary αMouse-HRP (1:3000, Dako 2016-09) or αRabbit-HRP (1:3000, Dako 2017-04) was detected with ECL Western blotting detection reagents (GE Healthcare RPN2106) on blue-sensitive film (CEA).

### Data deposition

All data files have been submitted and are available at PlasmoDB (http://www.plasmodb.org) and will be available at Gene Expression Omnibus (reference number: GSE39702).
