# Supplementary material for: H2A.Z/H2B.Z double-variant nucleosomes inhabit the AT-rich promoter regions of the Plasmodium falciparum genome
Source: Mol Microbiol. 2013 Jan 28;87(5):1061–73. doi: 10.1111/mmi.12151 (PMC3594968; doi:10.1111/mmi.12151)
Supplement: Supplementary file 1 [file mmi0087-1061-SD1.pdf]

SUPPLEMENTARY INFORMATION

for

H2A.Z/H2B.Z double-variant nucleosomes inhabit the AT-rich promoter regions of the *Plasmodium falciparum* genome

Wieteke A.M. Hoeijmakers[1], Adriana M. Salcedo-Amaya[1], Arne H. Smits[1,#], Kees-Jan Françoijs[1], Moritz Treeck[2,^], Tim-Wolf Gilberger[2,3], Hendrik G. Stunnenberg[1,] and Richárd Bártfai[1,*]*

1, Department of Molecular Biology, Radboud University, Nijmegen Centre for Molecular Life Sciences, Nijmegen, 6525GA,The Netherlands
2, Malaria Group, Bernhard-Nocht-Institute for Tropical Medicine, Hamburg, 20359, Germany
3, Pathology and Molecular Medicine, M.G. DeGroote Institute for Infectious Disease Research, McMaster University, Hamilton, ON, L8S 4K1, Canada

[*] To whom the correspondence should be addressed to: Richárd Bártfai or Henk Stunnenberg
Department of Molecular Biology, 274 NCMLS, P.O. Box 9101, 6500 HB Nijmegen, The Netherlands
tel: +31-24-3610523; fax:+31-24-3610520
email: R.Bartfai@ncmls.ru.nl or H.Stunnenberg@ncmls.ru.nl

Contains Supplementary Figures S1-5.

Supplementary Fig. S1:

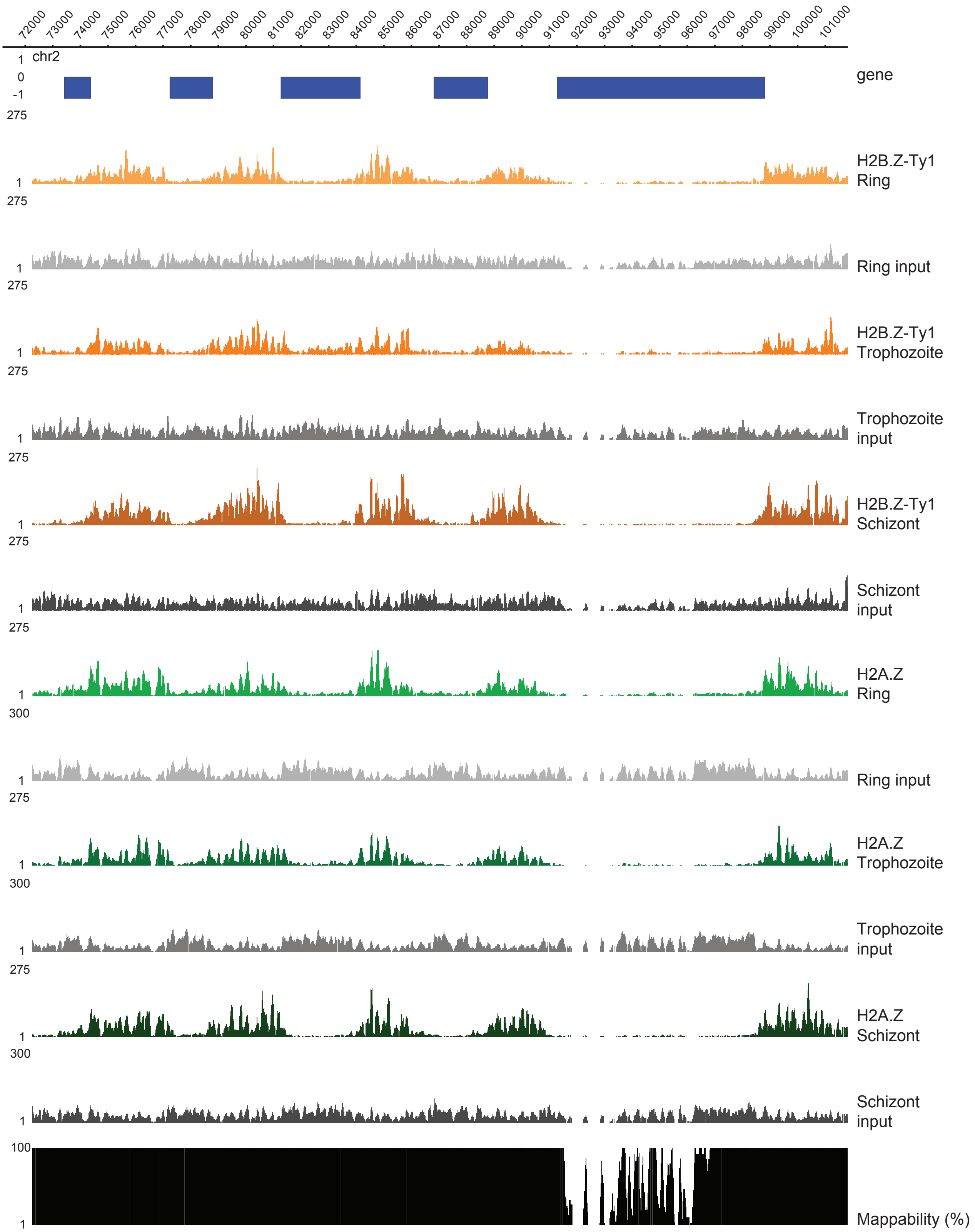

Supplementary Fig. S1: *Pf*H2A.Z and *Pf*H2B.Z have similar genome-wide localization profiles throughout intraerythrocytic development and reside in the same nucleosome particle. Screenshot of the H2B.Z-Ty1 and H2A.Z ChIP-Seq coverage plots as well as corresponding input tracks over a part of a euchromatic region of *P. falciparum* chromosome 2 at ring, trophozoite and schizont stage parasites (the same region is displayed in Fig.3). Genes are depicted as blue boxes and a 76bp sequence-read mappability track is included in black. Note that some variation between H2B.Z-Ty1 and H2A.Z input tracks can be observed. This is the consequence of variable sequence bias from Illumina version v4 and v2/v3 cluster generation and sequencing kits which were used to generate H2B.Z-Ty1 and H2A.Z input tracks, respectively, highlighting the importance of using corresponding ChIP and input tracks for analysis.

Supplementary Fig. S2:

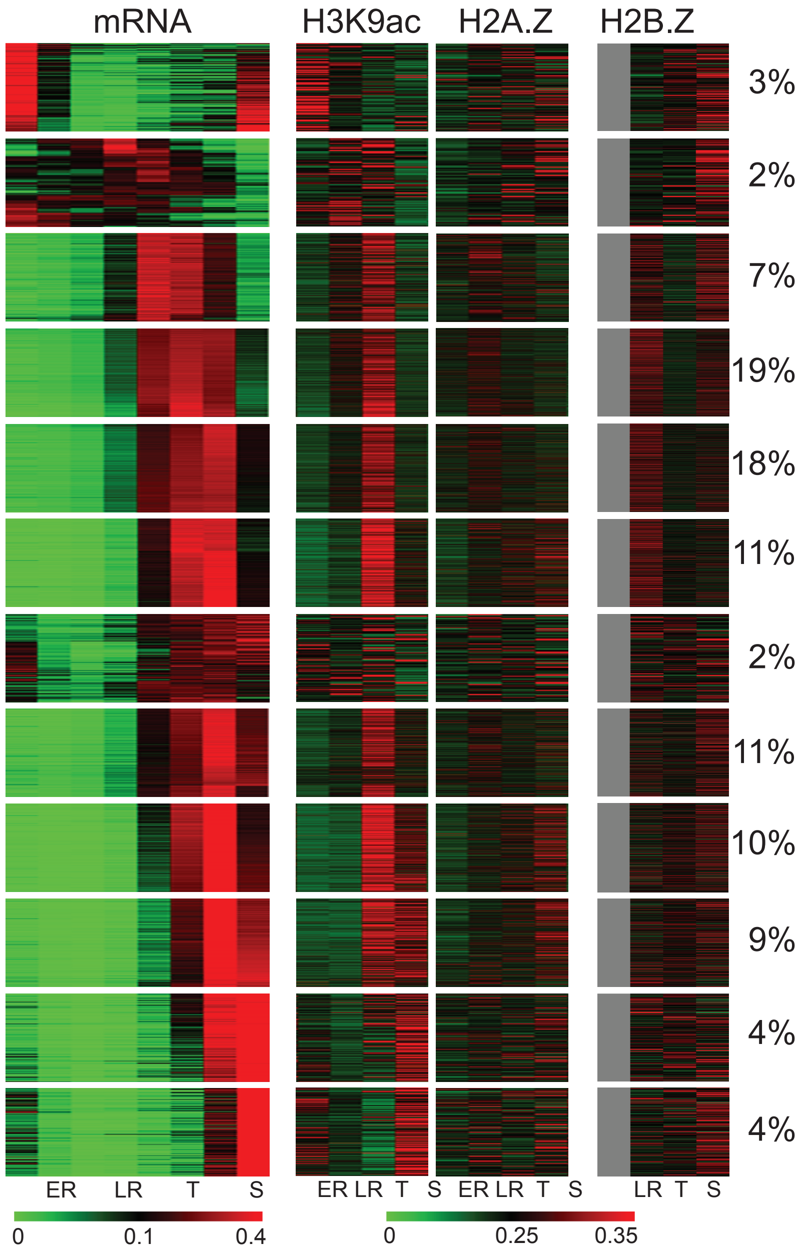

Supplementary Fig. S2: *Pf*H2A.Z and *Pf*H2B.Z occupancy does not change with temporal promoter activity.

Heatmap representation of the relative transcriptional activity of ~3800 euchromatic genes from (Bartfai et al., 2010) and the relative H3K9ac (from Bartfai et al., 2010), H2A.Z (from Bartfai et al., 2010) and H2B.Z marking upstream of genes throughout intraerythrocytic development (1 equals the sum of total occupancy values at all stages; H2B.Z values were adjusted for the lack of early ring data). K-means clustering of mRNA data was performed after correcting for total transcriptional activity per nucleus (Supplementary Figure S4 from Bartfai et al., 2010) and resulted in 12 groups of genes with different expression profiles. The percentage of genes belonging to each cluster is indicated on the right.

Supplementary Fig S3:

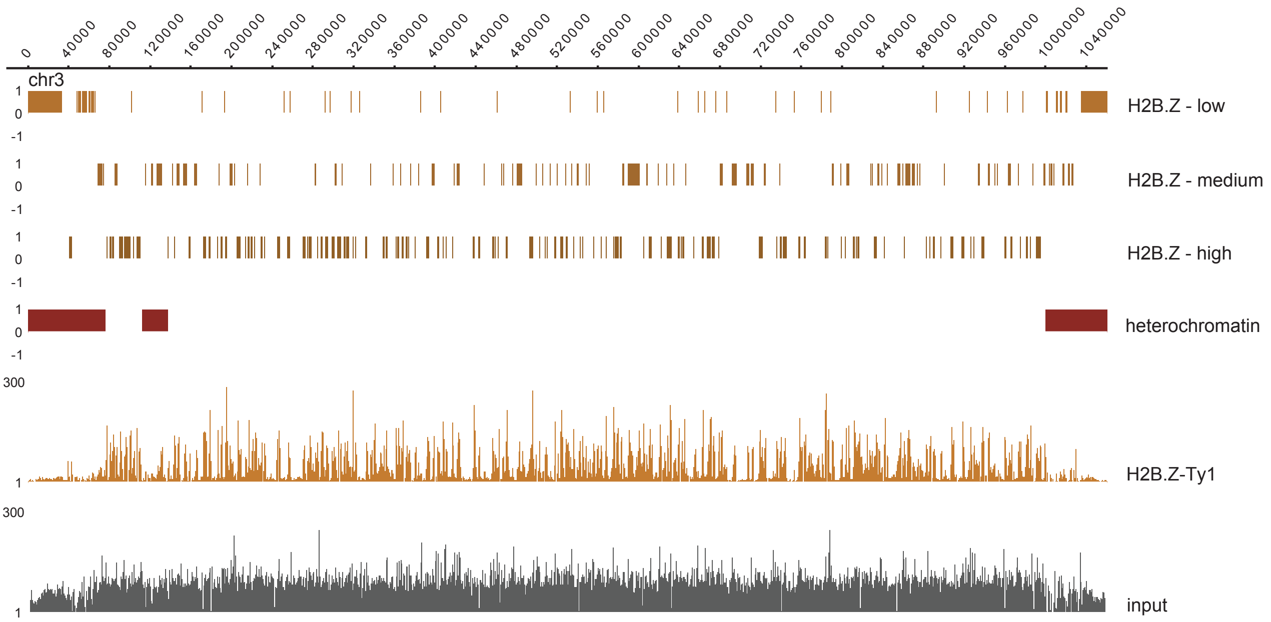

Supplementary Figure S3: *P. falciparum* intergenic regions divided based on low, medium or high H2B.Z occupancy levels localize to distinct genomic locations. Localization of H2B.Z-Ty1-low, -medium and -highly occupied regions (from Figure 4A) over *P. falciparum* chromosome 3. Heterochromatic regions, the H2B.Z-Ty1 as well as the input coverage plot are included at the bottom.

Supplementary Fig. 4:

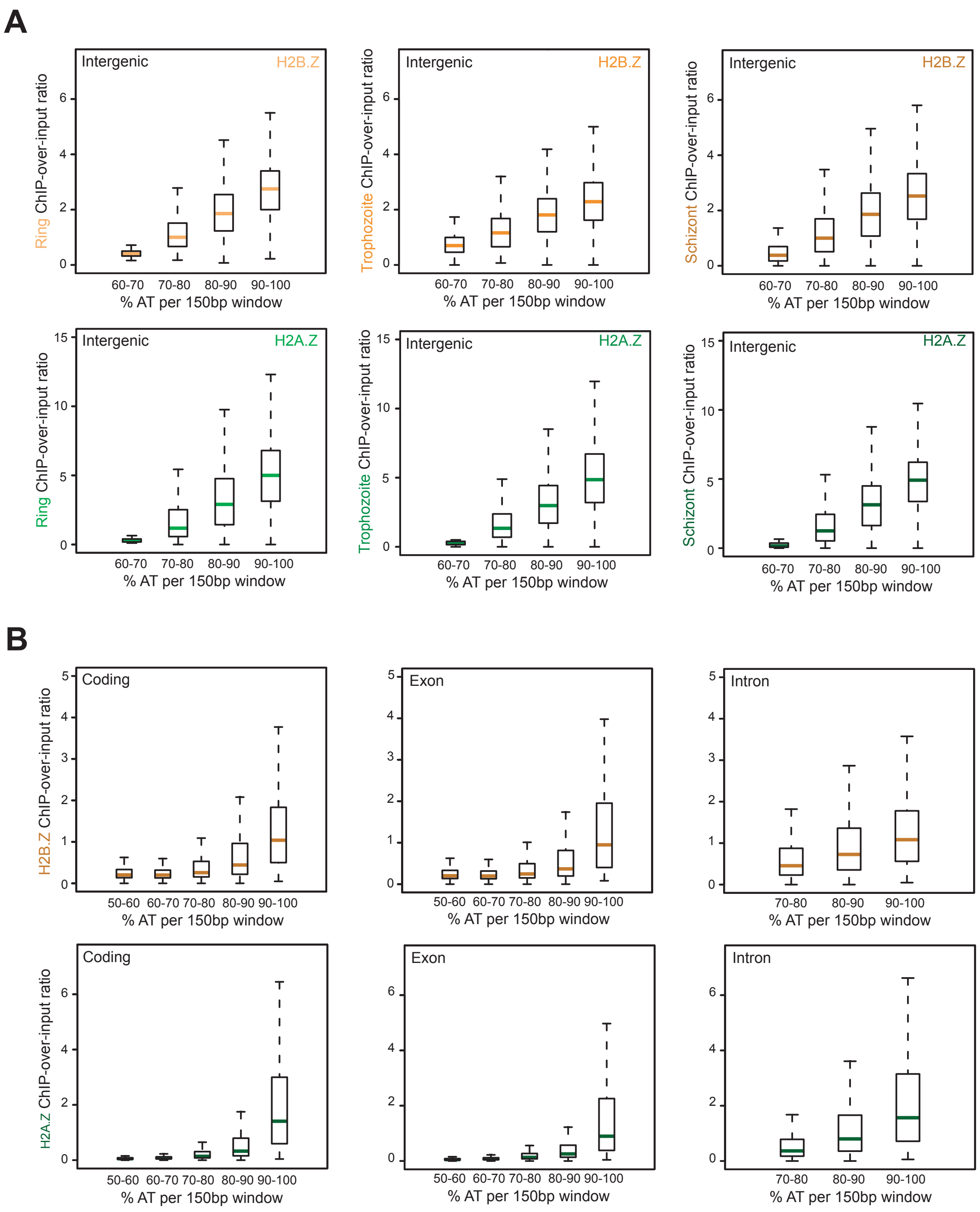

Supplementary Fig. S4: *Pf*H2B.Z and *Pf*H2A.Z occupancy increases with genomic AT-content. Box-plots displaying the distribution of H2B.Z-Ty1 or H2A.Z ChIP-over-input ratio's in 150bp windows of (**A**) euchromatic intergenic regions plotted against their genomic AT-content for ring, trophozoite and schizont stage derived libraries and (**B**) euchromatic coding, exonic and intronic regions plotted against their genomic AT-content for schizont stage derived histone variant libraries.

Supplementary Fig S5:

Max expression (tag/kb)	AT% (Avg+-SD)	# genes
0-10	84,8 +- 1,7	53
10-100	85,7 +- 2,1	242
100-1000	87,1 +- 2,2	1138
1000-10000	88,6 +- 2,2	391
10000-100000	89,9 +- 2,4	31

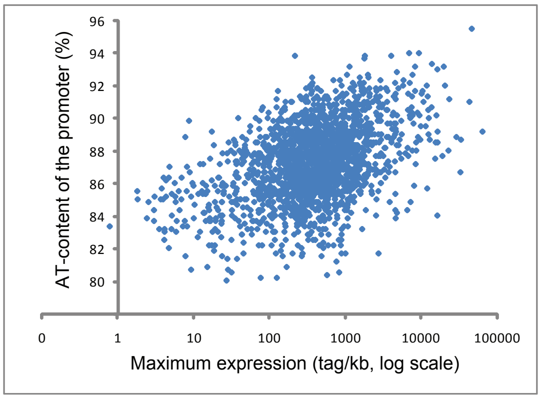

Supplementary Figure S5: Moderate but clear trend between AT-content of promoter regions and their strength. Table displaying the average AT-content of promoters with different maximal activity during intraerythrocytic development (maximum number of RNAseq reads per kilobase of coding sequence during 8 stages of asexual development (Bartfai et al., 2010)). The graph depicts the same data in form of a scatter plot.
